# Anti-Gamma-Aminobutyric Acid B (GABA-B) Receptor Encephalitis: A Case Series Analyzing Treatment Modalities and Cancer Screening Recommendations

**DOI:** 10.7759/cureus.90318

**Published:** 2025-08-17

**Authors:** Sebastian Garza Hernandez, Hayden Johnson, Binod Wagle, Nooshin Kiani Nia

**Affiliations:** 1 Department of Neurology, University of Missouri-Kansas City School of Medicine, Kansas City, USA; 2 Department of Neurology, University of Cincinnati Health, Cincinnati, USA

**Keywords:** anti-gaba-b receptor antibodies, autoimmune encephalitis, cancer screening strategy, immunotherapy, status epilepticus (se)

## Abstract

This case series presents an alternative treatment approach for refractory epilepsy in the setting of anti-gamma-aminobutyric acid B receptor (GABA-B-R) encephalitis and examines current guidelines for long-term cancer screening.

We present two cases of anti-GABA-B receptor encephalitis from Kansas City, Missouri. The first case describes a 53-year-old woman presenting with new-onset headache, seizures, and progressive encephalopathy, who was treated with corticosteroids and anti-seizure medications, followed by intravenous immunoglobulin (IVIG) and plasma exchange (PLEX). Cancer screening with chest CT was negative. Fluorodeoxyglucose (FDG)-PET revealed increased uptake in two cervical lymph nodes; however, her biopsy was negative for malignancy. The second case describes a 49-year-old woman presenting with confusion, dizziness, and seizures. Her seizures were refractory to anti-seizure medications and immunotherapy, but an excellent response and recovery were seen after the concurrent use of methylprednisolone, IVIG, and anti-seizure medications. Subsequent malignancy screenings remained negative in both cases.

A combination of corticosteroids with IVIG or PLEX may be a promising option for refractory cases of anti-GABA-B-R autoimmune encephalitis (AE). Additionally, it is not clear whether malignancies other than small cell lung carcinoma (SCLC) are strongly associated with this condition. Further studies are needed to evaluate the option of combined immunotherapy and the association of other malignancies with anti-GABA-B AE.

## Introduction

Viral, fungal, or bacterial infections are typically understood as primary culprits of encephalitis; however, more esoteric causes include postinfectious, paraneoplastic, idiopathic, or autoimmune-mediated inflammatory processes. The study of autoimmune encephalitis is a rapidly expanding condition with numerous subtypes and varying theories in the current literature. Anti-gamma aminobutyric acid B receptor (GABA-B-R) encephalitis is a specific subgroup of autoimmune encephalitis (AE), which was first described in the literature in 2010 by Lancaster et al. [1].

The primary symptoms of anti-GABA-B-R encephalitis are seizures, limbic dysfunction, cognitive symptoms, and psychiatric manifestations. Diagnosis is based on the clinical presentation, neuroimaging including brain MRI, EEG findings, and cerebrospinal fluid (CSF) studies with autoantibody testing. CSF PCR and culture are useful for excluding infectious etiologies of encephalitis, which are often managed empirically with antibiotics and antivirals until ruled out [1-4]. Common signs include limbic dysfunction or temporal lobe pathology on MRI and EEG [2]. Unfortunately, anti-GABA-B receptor encephalitis has a high mortality rate within five years [5]. Current treatment involves immunotherapy, chemotherapy, or plasmapheresis [3].

A strong correlation with underlying small cell lung carcinoma (SCLC) has also been found, but there is uncertainty in long-term cancer screening [1-4]. Additional studies are emerging regarding identifying cases associated with malignancy with the new additional subgroup of antibodies, such as the potassium channel tetramerization domain containing 16 (KCTD16) [5].

We describe the clinical course, treatment responses, and malignancy screening approaches in two patients diagnosed with anti-GABA-B-R encephalitis presenting with refractory seizures. In addition, we discuss the available treatment modalities and long-term cancer screening recommendations for these type of patients.

## Case presentation

Case 1

**Figure 1:**
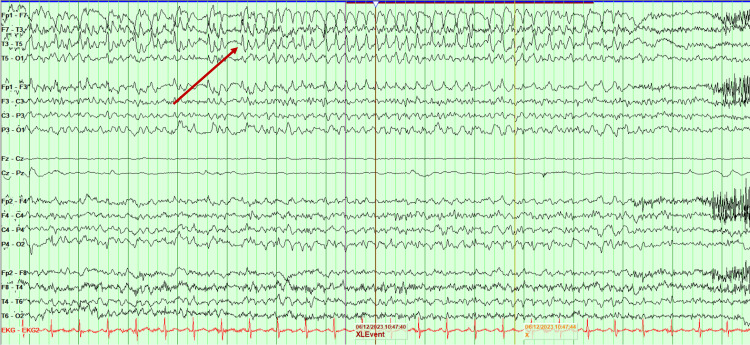
EEG study capturing an electrographic seizure Case 1 EEG capturing subclinical seizures that were all self-limited, typically lasting less than 60 seconds (arrow). No observable clinical behavioral change was seen with any of these seizures. The seizures appeared to have a focus of onset over the left temporal lobe, and there were frequent sharp discharges seen over the left anterior temporal region

A 53-year-old previously healthy Hispanic woman presented with new-onset headache, seizures, and progressive decline in mentation for the last several weeks. On neurologic examination, her mentation was fluctuating daily; however, she was often somnolent or agitated. She was disoriented, had memory impairment, and had trouble following commands, in addition to emotional incontinence. Reflexes were diffusely increased. MRI was unremarkable, except for an incidental small chronic right cerebellar lacunar infarct. She had multiple EEGs significant for subclinical seizures originating from the left temporal region, in addition to rare sharp discharges (Figure 1). CSF analysis showed a lymphocytic dominant white blood cell (WBC) count of 53/mm^3^, red blood cell (RBC) count of 381/mm^3^ without xanthochromia, protein of 30 mg/dL, and glucose of 60 mg/dL. CSF meningitis/encephalitis PCR was negative. CSF autoimmune/paraneoplastic panel was positive for anti-GABA-B-R antibodies with titers at 1:256. The patient was treated with aggressive immune-targeted treatment, including methylprednisolone, plasma exchange (PLEX) therapy, and intravenous immunoglobulin (IVIG). Additionally, she required multiple antiseizure medications (ASMs) to control her seizures.

**Figure 2:**
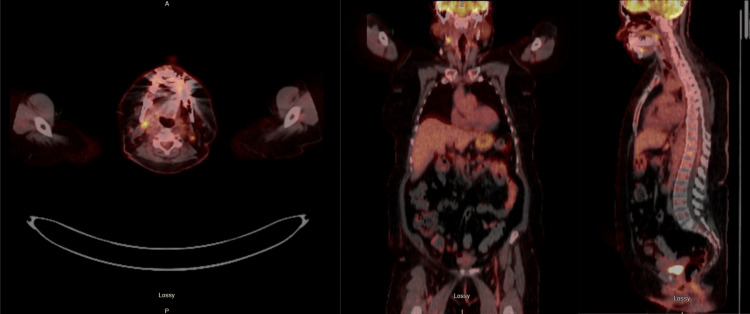
Case 1 FDG-PET CT of the skull base to the thigh Case 1 FDG-PET CT of the skull base to the thigh showed indeterminate FDG-avid left greater than the right cervical chain lymph nodes FDG: fluorodeoxyglucose

PET scan showed increased fluorodeoxyglucose (FDG) uptake in two cervical lymph nodes (Figure 2). A CT of her neck showed submandibular lymphadenopathy and abnormal oral soft tissue. Two subsequent lymph node biopsies had unrevealing pathology. The seizures were successfully controlled, and the mental status gradually improved. She was noted to be back to her baseline neurologic status at her 16-month follow-up appointment and continues to be monitored for occult malignancy with regular screening images.

Case 2

**Figure 3:**
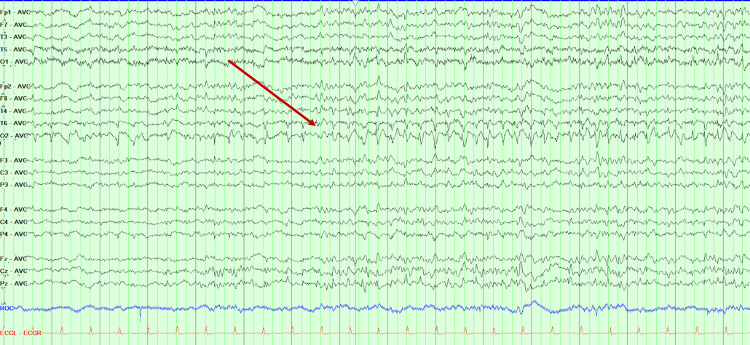
EEG capturing an electrographic seizure Case 2 EEG capturing multiple electrographic seizures in the right temporal-occipital area. The electrographic seizure consists of spike and slow wave starting in the O2 lead and subsequently 4-5 Hz rhythmic activity with some spread to the T6 lead (arrow)

**Figure 4:**
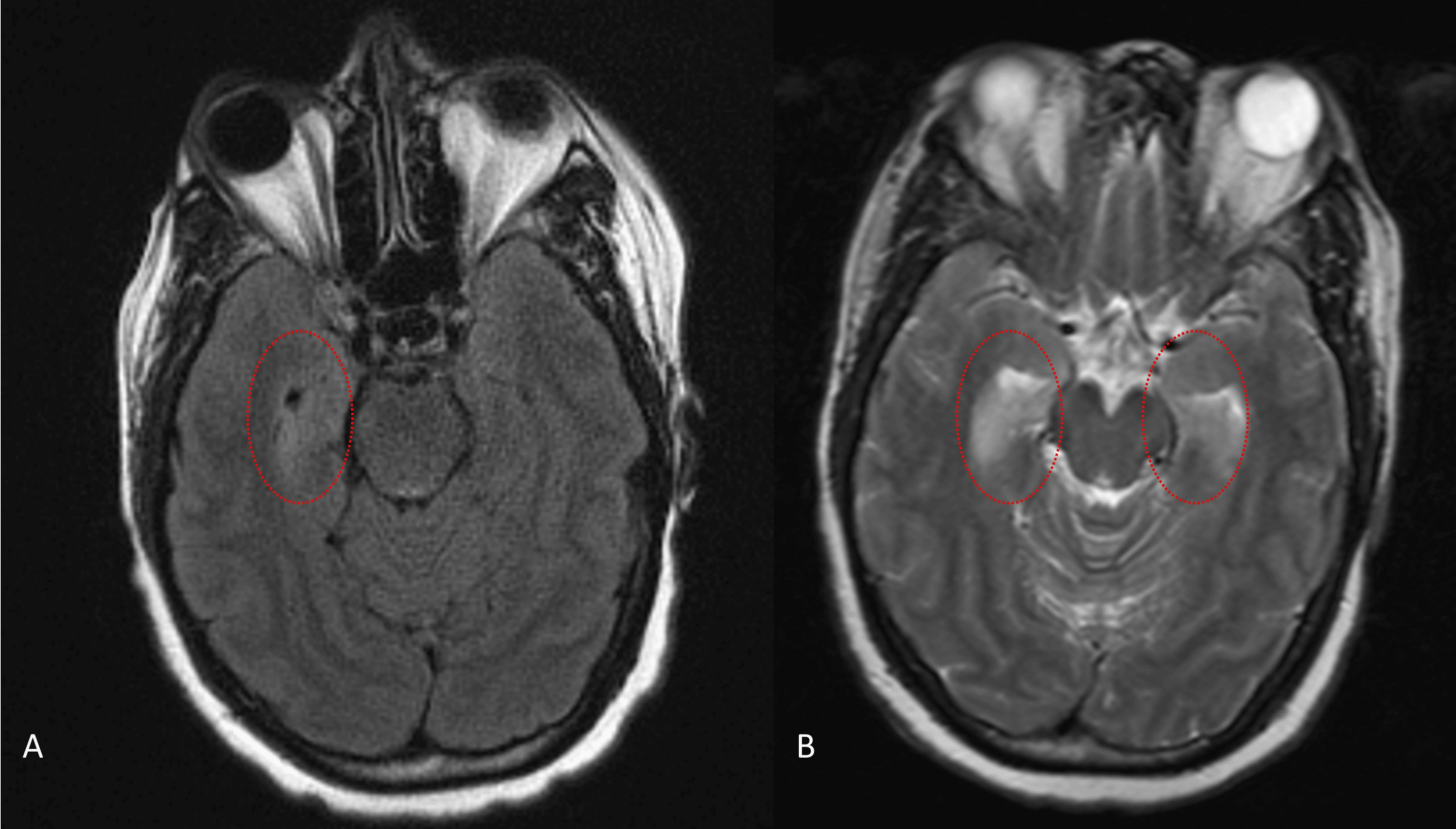
Case 2 brain MRI with signs of AE (A) Case 2 initial brain MRI axial view of the T2 FLAIR sequence revealing hyperintensity involving the right mesial temporal lobe. (B) Case 2 subsequent brain MRI axial view of the T2 FLAIR sequence revealing worsening hyperintensity involving bilateral mesial temporal lobes, suggestive of limbic encephalitis AE, autoimmune encephalitis; FLAIR, fluid-attenuated inversion recovery

A 49-year-old woman presented with two days of confusion, dizziness, and short-term memory loss, as well as mild behavioral changes in the last few weeks. On initial presentation, she was hypertensive and disoriented, but physical examination, routine laboratory tests, and head CT angiography were unremarkable. She was discharged home; however, she returned the following day for worsening confusion and seizures. During admission, her seizures became more frequent, and EEG confirmed refractory focal status epilepticus originating from the right temporal-occipital area despite the use of multiple ASMs (Figure 3). Brain MRI revealed progressively worsening T2 signals in the right mesial temporal lobe, which later progressed to bilateral mesial temporal and occipital lobes (Figure 4). The CSF analysis was significant for WBC of 80/mm^3^, RBC of 22/mm^3^, and protein of 25 mg/dL. A CSF BioFire test was negative for infectious causes of encephalitis. The CSF autoimmune panel resulted in positive for anti-GABA-B-R autoantibodies with a titer of 1:1024.

Her treatment for AE included methylprednisolone for five days and then five sessions of plasmapheresis every other day. Despite treatment, there was no improvement in the subclinical electrographic seizure, and the brain MRI lesions worsened. Eventually, the patient was started on a combination of IVIG and methylprednisolone due to continued electrographic seizures. Within 24 hours after starting the combination therapy, the seizure frequency improved. The patient became seizure-free following five days of combination therapy and was eventually discharged. Three and a half years later, the patient is on no medications, reports minimal cognitive impairments, and can live and function independently. Ongoing SCLC screening is still negative for malignancy.

## Discussion

Anti-GABA-B-R encephalitis is a rare type of AE, with an abnormal immune response due to antibodies against GABA-B receptors. These antibodies block the receptor function, and because GABA-B is an inhibitory neurotransmitter, the neuronal excitability increases, leading to decreased seizure threshold. There is a close relationship with SCLC, and in these cases, patients usually will not have a good response to immunotherapy, carrying a poor prognosis [6].

First-line immunotherapy for anti-GABA-B receptor encephalitis includes corticosteroids, IVIG, or PLEX. Corticosteroids in combination with either IVIG or PLEX, while less common, may be an effective approach for refractory cases, as shown in the second case. Rituximab and cyclophosphamide are two of the more commonly used second-line agents for patients who are unresponsive or unable to take first-line therapies [7]. In patients found to have a primary tumor, early tumor therapy is paramount in improving their prognosis. For the treatment of seizures, ASMs should be used in combination with proper immunotherapy. ASMs are often stopped after clinical recovery and a prolonged seizure-free period, although long-term maintenance therapy should be considered for patients with permanent brain damage or mesial temporal lobe atrophy [8].

According to the European Federation of Neurological Societies (EFNS), screening should focus on the organ suggested by the antibody and clinical symptoms. GABA-B receptor AE is primarily associated with SCLC in 60% of the cases, and in these patients, CT of the chest, followed by FDG-PET, is the recommended screening. If the initial screening is negative, screening should be repeated after 3-6 months, followed by every six months for 2-4 years. However, there is no recommendation regarding screening if there is no concern for malignancy in the organ most associated with that antibody [9]. In a recent study, the co-occurrence of the KCTD16 antibodies points toward a paraneoplastic origin, which might help with the screening decision [5]. In Case 1, the initial chest CT scan was negative for malignancy, but FDG-PET revealed an increased uptake in two cervical lymph nodes from which a biopsy was obtained to screen for malignancy; however, the results remained negative. It was assumed to be a false positive or an incidental finding. In Case 2, CT of the chest, abdomen, and pelvis was performed on multiple occasions as cancer screening for two years.

Early aggressive immunotherapy is warranted after the suspicion of anti-GABA-B-R AE, given the high potential for recovery and risks associated with delayed treatment. Immunotherapy is the mainstay of treatment, as most patients experience improved neurologic functioning and better long-term outcomes [10]. While first-line immunotherapy includes corticosteroids, IVIG, or PLEX, a combination of corticosteroids with IVIG or PLEX may be promising for refractory cases [7]. ASMs should be combined with immunotherapy to treat associated seizures, and tumor therapy should be initiated if a source of malignancy is found [8].

## Conclusions

It is not clear whether malignancies other than SCLC are strongly associated with anti-GABA-B-R AE. As a result, when initial screening for malignancy is unrevealing, it is controversial to screen for other malignancies. Additionally, although it will be inappropriate to miss an occult malignancy, excessive testing and biopsies may cause patients and families a significant economic and psychological burden, as seen in Case 1. Further standardized guidelines are needed for malignancy screening in the setting of AE. Biomarkers, such as KCTD16 antibodies, might help in establishing these recommendations.

The good treatment response in Case 2 raises the question of whether further studies should evaluate the option of combined immunotherapy and the association of other malignancies with anti-GABA-B-R AE.
